# Functional and aesthetical full‐mouth rehabilitation of a patient with severely worn dentition and deep bite using minimally invasive approach in current vertical dimension: A 3‐year follow‐up

**DOI:** 10.1002/ccr3.8257

**Published:** 2023-11-27

**Authors:** Negin Yaghoobi, Azam Mostafavi, Solmaz Barati

**Affiliations:** ^1^ Dental Research Center, Dentistry Research Institute, Department of Prosthodontics Tehran University of Medical Sciences Tehran Iran

**Keywords:** bruxism, computer‐aided design, full‐mouth reconstruction, tooth wear

## Abstract

**Key Clinical Message:**

Regaining restorative space for fullmouth reconstruction in a patient with deep bite and worn dentition by conservative approach in current vertical dimension and also using minimally invasive concept is recommended.

**Abstract:**

Full mouth reconstruction of a deep bite patient with severely worn dentition is a challenging situation for the prosthodontists. This study represents minimally invasive procedures in mentioned condition without increasing vertical dimension. After 3 years of follow up no complication was observed.

## Graphical abstract

Regaining restorative space for full mouth reconstruction in a patient with deep bite and worn dentition is a challenging situation. Since an alteration in VDO may cause adaptable reactions in the temporomandibular joint (TMJ), periodontium, and occlusal morphology, regaining restorative space by conservative approach in current vertical dimension and also using minimally invasive concept is recommended.

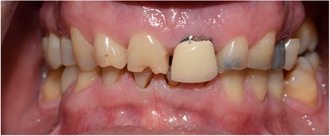

## 1 INTRODUCTION

Tooth surface loss (TSL) or tooth wear (TW) is an irreversible loss of hard teeth structure which is observed clinically as attrition, abrasion, abfraction, and erosion.^1^ Different types of tooth wear frequently coexist, making it difficult to determine the type of wear present.^2^

Abrasion is defined as physical wear of the teeth caused by something other than tooth‐to‐tooth contact like inappropriate toothbrushing or repeated use of a toothpick.^2^ Furthermore, the loss of tooth structure may occur when the teeth are in contact with one another, as a result of attrition or dental erosion caused by acids in the mouth. An abfraction, a wedge‐shaped lesion with sharp line angles, has been attributed to eccentric forces acting on the natural dentition,^3^ whereas Dawson believed it is the result of tooth brushing rather than occlusal overload.^4^, ^5^

Severe tooth wear could cause tooth hypersensitivity related to dentine exposure, morphological change of occlusal surfaces of teeth, occlusal disharmony, functional impairment, temporomandibular joint disorders (TMD), and also poor aesthetic.^1^, ^6^, ^7^ In addition, loss of occlusal vertical dimension (OVD) may lead to dentoalveolar compensation or increased interocclusal rest space.^8^

Due to progress in restorative methods and modified materials there are many treatment options for patients with worn dentition such as: conventional full coverage restorations, direct or indirect composite resin restorations, cast adhesive alloys (metal palatal veneers), and bonded ceramic restorations.^9^

Malocclusion, which can be defined as “an abnormal occlusion in which teeth are out of alignment with adjacent teeth in the same jaw or the opposing teeth when the jaws are closed”,^3^ can result in oral health complications if left untreated. As a specific type of malocclusion, deep bite is defined as an increased vertical overlap between the upper and lower incisors which is treated by several methods range from removable appliances to fixed appliances with or without orthognathic surgery^10^; It is, however, believed that deep overbite with stable holding contacts constitute one of the most stable dentitions, in which case no dental treatment is necessary.^5^

Although restoration of worn teeth by reorganizing the occlusion at an increased VDO has been well documented, some complaints, such as TMD, chipping of unsupported ceramics, and relapse to previous conditions are frequent.^6^, ^7^, ^11^ For worn teeth with insufficient restorative space, intentional endodontic treatment, crown lengthening, and full coverage restorations have been recommended.^5^, ^12^

While full coverage restorations have been recommended to restore worn teeth with insufficient restorative space, based on the available literature, conservative approaches such as minimally invasive restorations have been found to be more conservative than conventional procedures.^13^, ^14^

A few examples of minimally invasive approaches that might be indicated in such worn dentition include establishing occlusal harmony with an ideal occlusal plane through laminate veneer restorations, as well as occlusal veneers.^15^, ^16^, ^17^

Consequently, a full‐mouth rehabilitation involving an interdisciplinary approach for a patient with severe deep bite and worn dentition is described in this clinical report.

## 2 CASE PRESENTATION

**FIGURE 1:**
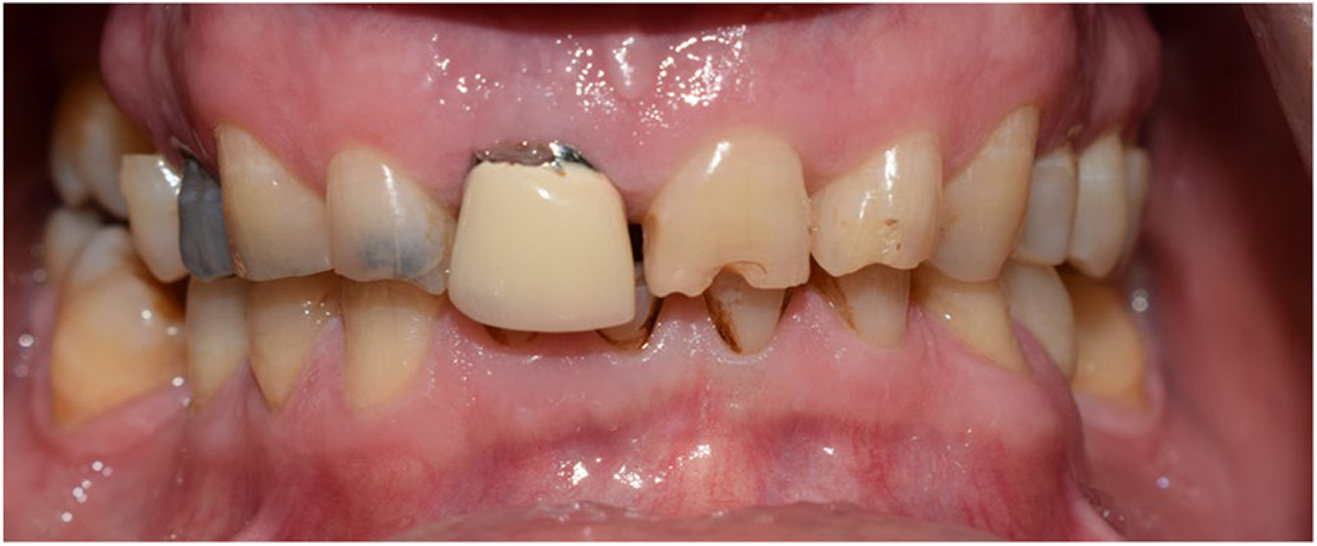
Intraoral frontal view; beginning of treatment 2018.

A 54‐year‐old man was referred to the department of prosthodontics for oral rehabilitation. He had informed consent to present his treatment approach and also his photographies. His chief complaint was being worry about worn dentition and poor esthetic especially in anterior teeth. There was no specific diet or mal habit mentioned by the patient other than clenching and bruxism during the night. He reported pain in his masticatory muscles when waking up and his partner mentioned hearing sound of teeth grinding when he was sleeping. Extraoral examination revealed almost symmetrical esthetic proportions and no limitations or deviation was observed during maximum mouth opening. palpation of muscles, lymph nodes, and temporomandibular joints confirmed normal conditions. Intraoral examination revealed uneven incisal plane, smile disharmony, and uneven gingival plane in both arches. General tooth surface loss and dentin exposure especially in mandibular incisors were detected without pain. (Figure 1).

**FIGURE 2:**
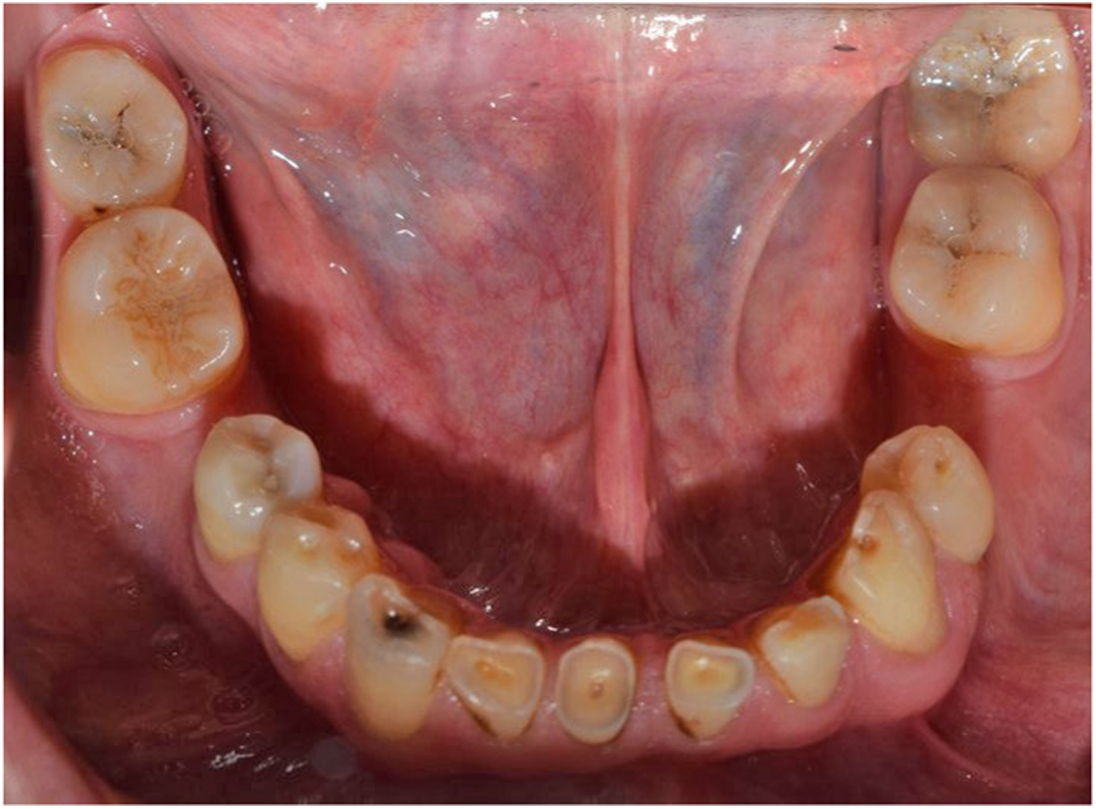
Mandibular occlusal view; beginning of treatment 2018.

**FIGURE 3:**
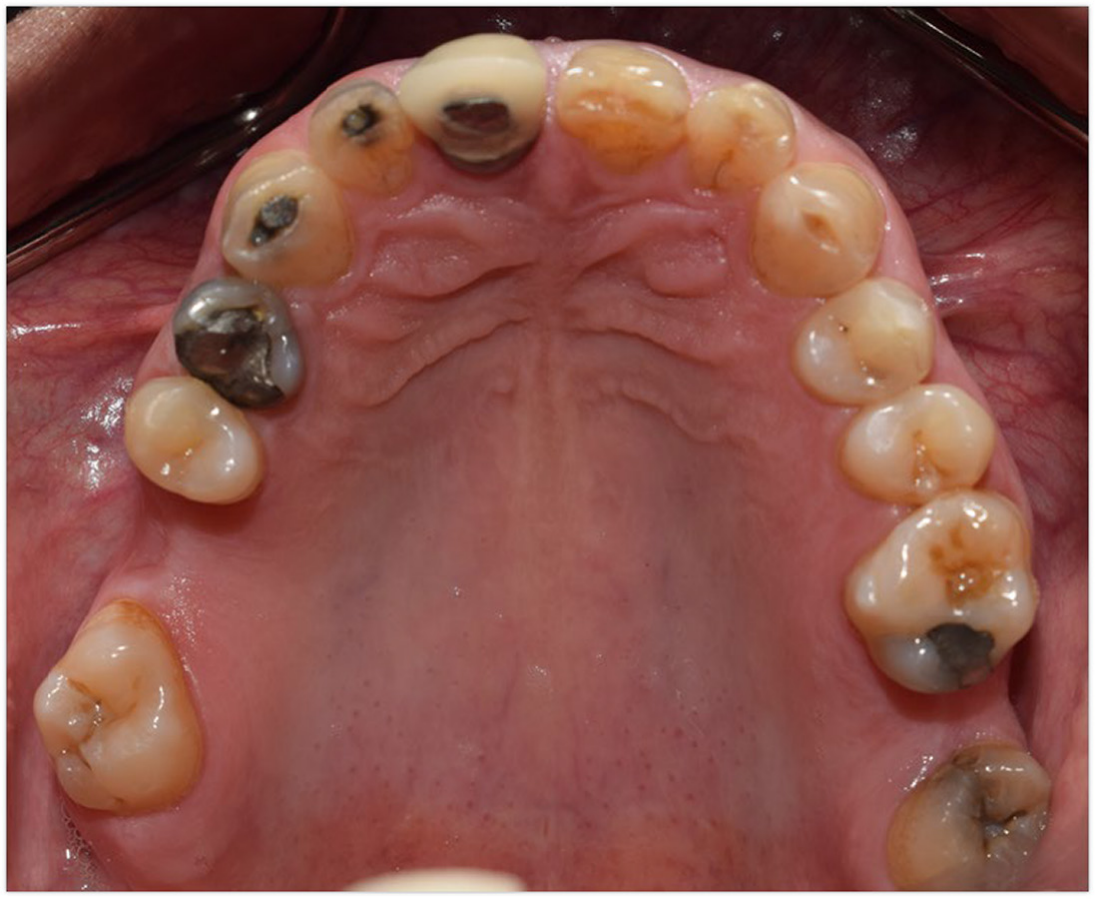
Maxillary occlusal view; beginning of treatment 2018.

**FIGURE 4:**
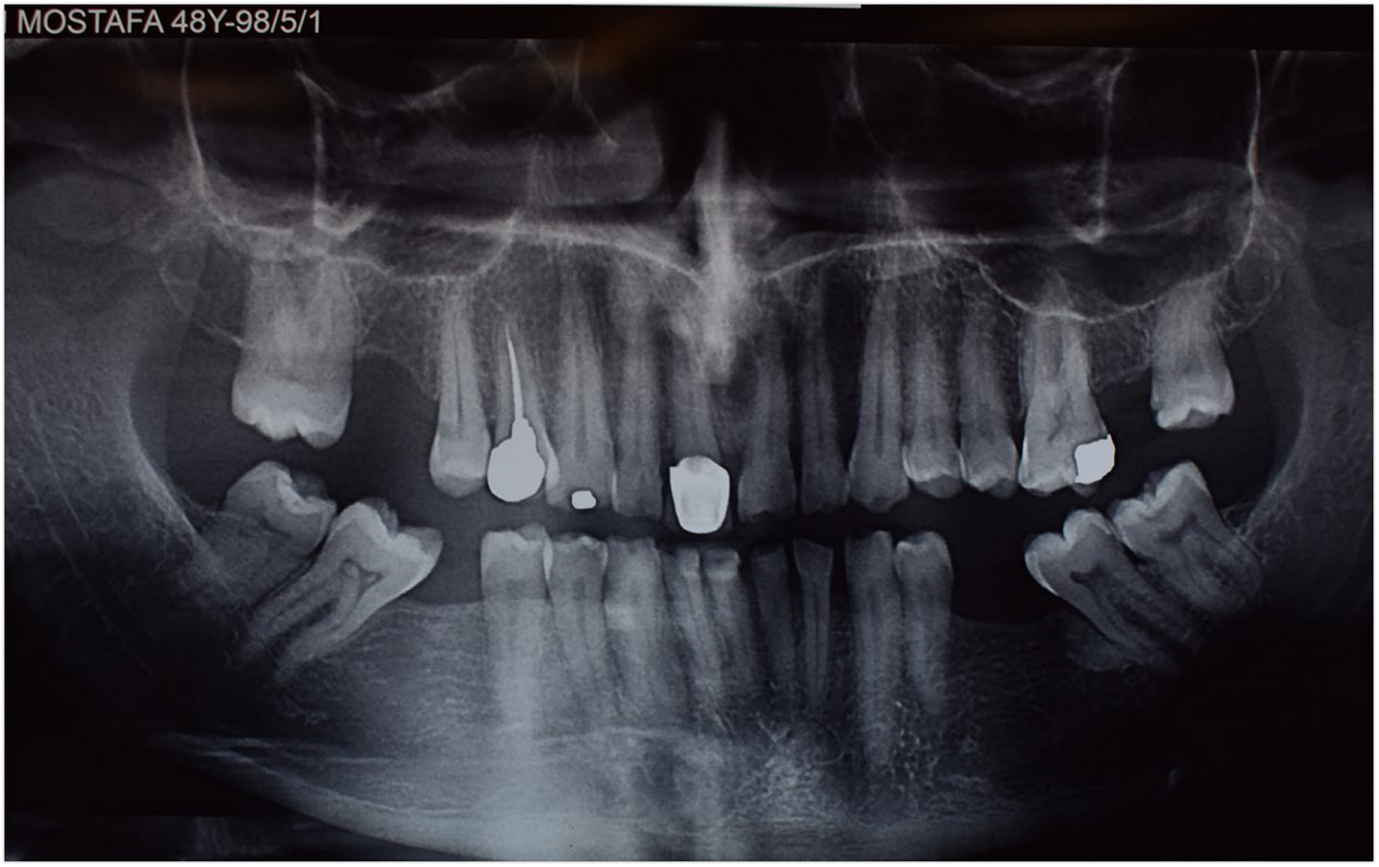
Initial panoramic view; beginning of treatment 2018.

Abfraction of teeth #4 and #5 and unilateral lingual torus mandibularis in right canine/premolar area was observed (Figure 2). Caries in teeth #3 #17#32 were detected. There was an amalgam filling in tooth number #5 that was not appropriate from both an aesthetic perspective and in terms of durability (Figure 3). In the first visit, tooth number 7 had a metal ceramic full crown which did not match the color of the other teeth, showed metal margin exposure and an incorrect contour, all of which were noted in the smile view. Missing teeth of number #19 #20 #30, mesiolpalatal tilt of tooth number 2, mesial drift of #18 and #31 that caused insufficient space for replacement of missing teeth and also disharmonic occlusal plane was evident. Oral hygiene was appropriate but there was insufficient attached mucosa in teeth number #18 #31. All teeth had good crown root ratio in radiographic periapical view except #16. Tooth number #5 had unacceptable RCT (Figure 4). VDO was evaluated and the freeway space was measured to be 4 mm (normal value is 2–4 mm).^18^ Dense bone island detected as localized area of radiopacity near mesial root of tooth #18. These localized, well‐defined, radiopaque lesions are asymptomatic and found more often in the mandible, especially in the molar region. Since their cause is unknown and their presence has no clinical significance, extraction of a tooth inserted into a DBI may result in an infected socket and bone resorption, therefore no specific treatment has been recommended.^19^ After related consultation, it was suggested that excess occlusal stress could be a contributing factor to DBI associated with the roots of the tooth, and that no treatment was indicated.

For the diagnostic stage, primary impressions one‐stage putty‐wash (speedex coltene/switzerland) were made and poured by plaster (Moldano Dental Stone, Bayer Co). Centric relation was recorded by bimanual manipulation technique using acrylic anterior deprogrammer (Pattern Resin LS, GC Dental Corp) and bite registration silicone (Futar D; Kettenbach GmbH & Co) as bite registration material. The record was used for mounting the primary casts in a semiadjustable articulator (Hanau Wide‐Vue Whip Mix) by an arbitrary facebow (Hanau Springbow‐Whip Mix). Posterior occlusion revealed interferences on teeth number #12, #13, #20, and #21. The possible treatment plan would be established according to investigation the space in vertical dimension at rest and occlusion and the space available for teeth restorations. Based on speech, smile evaluation, and amount of restorative space in CR position it was decided to rehabilitate the dentition in the existing VDO. As a result, VDO was considered in CR at the interference point, which was approximately a 0.5 mm opening in the posterior segment and a 1 mm increase in the anterior one. Wax‐up for lower anterior teeth was carried out after determination of mandibular canine level at the corner of the resting lips, followed by upper anterior teeth waxing. The quality and correctness of wax‐up were verified by chairside mock‐up (temporary crown and bridge material, master‐dent, USA) in the mouth during phonetics, smile, and rest position. Then occlusal plane was determined using a Broadrick occlusal plane analyzer and that was verified in mouth in centric occlusion and eccentric movements. It is important to mention that mutually protection occlusal scheme was considered. Based on occlusal plane, inserting implant to replace teeth #3 and #15, RCT for teeth #8 #24 #25 #26, re‐RCT of #5, crown lengthening of maxillary and mandibular anterior segment (from canine to canine), full crown of #4 #6 #8 #11, post and core crown #5 #23 #24 #25, fixed partial denture #18 #19 #20 and #29 #30 #31, porcelain laminate veneers of #7 #9 #10 #23, and occlusal veneers #12 #32 were decided.

**FIGURE 5:**
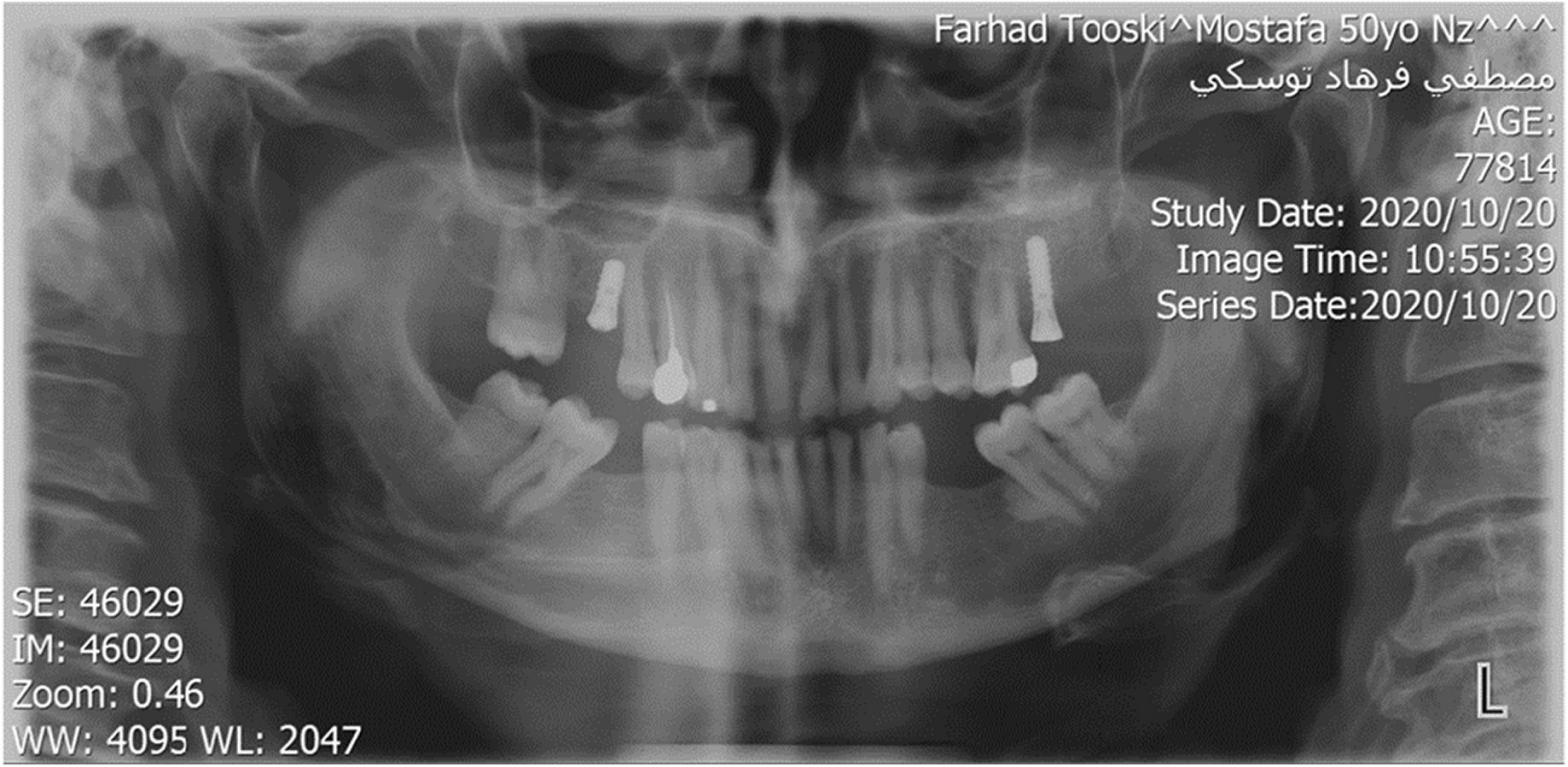
Panoramic view after implant surgery 2019.

Once the treatment plan was accepted, provisional restorations were made using temporary crown and bridge material (master‐dent, USA) for treatment sessions until the teeth preparation were finalized and printed temporary restorations were prepared. After scaling and root planning, caries removal was done and teeth were filled with composite (3 M™ Filtek™ Z350 XT Universal Restorative/USA). Crown lengthening stent for both jaws were made and gingival leveling in anterior segments of maxillary and mandibular arch was done in one session. Implant insertion was carried out using a semiguided surgical guide (radiographic stent converted to surgical stent) in location of teeth number #3 (Ø 4.8 mm RN, SLA® 8 mm Straumann® Dental Implant System), #15 (Ø 4.8 mm RN, SLA® 12 mm Straumann® Dental Implant System) (Figure 5). Fabrication of post resin pattern according to affirmed occlusal plane by index was done. Nickel‐chrome casting post was cemented by Glass ionomer luting cement (GC Fuji I®Enhanced America).

Preparation was done in this way: circumferential radial shoulder finish line was prepared for FPDs and crowns, light chamfer for laminates and conventional design (planar straight bevel) was considered for occlusal veneers.

Impressions for temporary restorations were taken (Betasil Vario Putty Soft Muller Germany/Betasil Vario Light Body Muller Germany); CR registration according to past method and facebow transfer and mounting was done. After abutment selection (straight and angled tissue level regular neck), temporary restorations design was done in Exocad DentalCAD software, then they were printed (DigiDent Lite 4 K, IRAN) by PMMA material shade A2 (kucco‐koul, China).

In try‐in session, smile view, occlusal plane, and occlusal contacts were assessed during CR and eccentric movements, and canine guidance and mutually protected occlusion was established. After assessing occlusion, marginal fitness and phonetic, impression making from temporary restorations and making Casts were done. Protrusive record for determining condylar inclination (R:30, L:26) and bennet angle (R:16, L:15) (using Hanau's formula: L = H/8 + 12) was completed and final impression from temporary restorations was taken. One‐stage putty‐wash (speedex coltene/switzerland) and customized occlusal table were prepared. Then, final Impression was made in the same way mentioned before. Casts were mounted by cross mount technique (every other mounting procedure), cores were designed using Exocad software, then frameworks were milled IPS e.max®ZirCAD (Ivoclar Vivadent, Germany) for PFZ crowns and IPS Empress CAD (Ivoclar Vivadent, Germany) for PLV and occlusal veneers.

Next, occlusal veneers were checked in mouth and radiographies were taken to check the marginal integrity. The porcelain was then applied to the frames (according to the manufacturer's instructions) and a porcelain try‐in session was conducted. After confirming restorations aesthetic and contours, final glaze and delivery was done.

**FIGURE 6:**
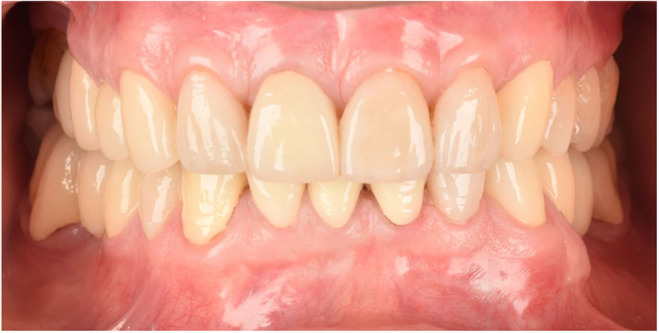
Intraoral frontal view after cementation 2019.

**FIGURE 7:**
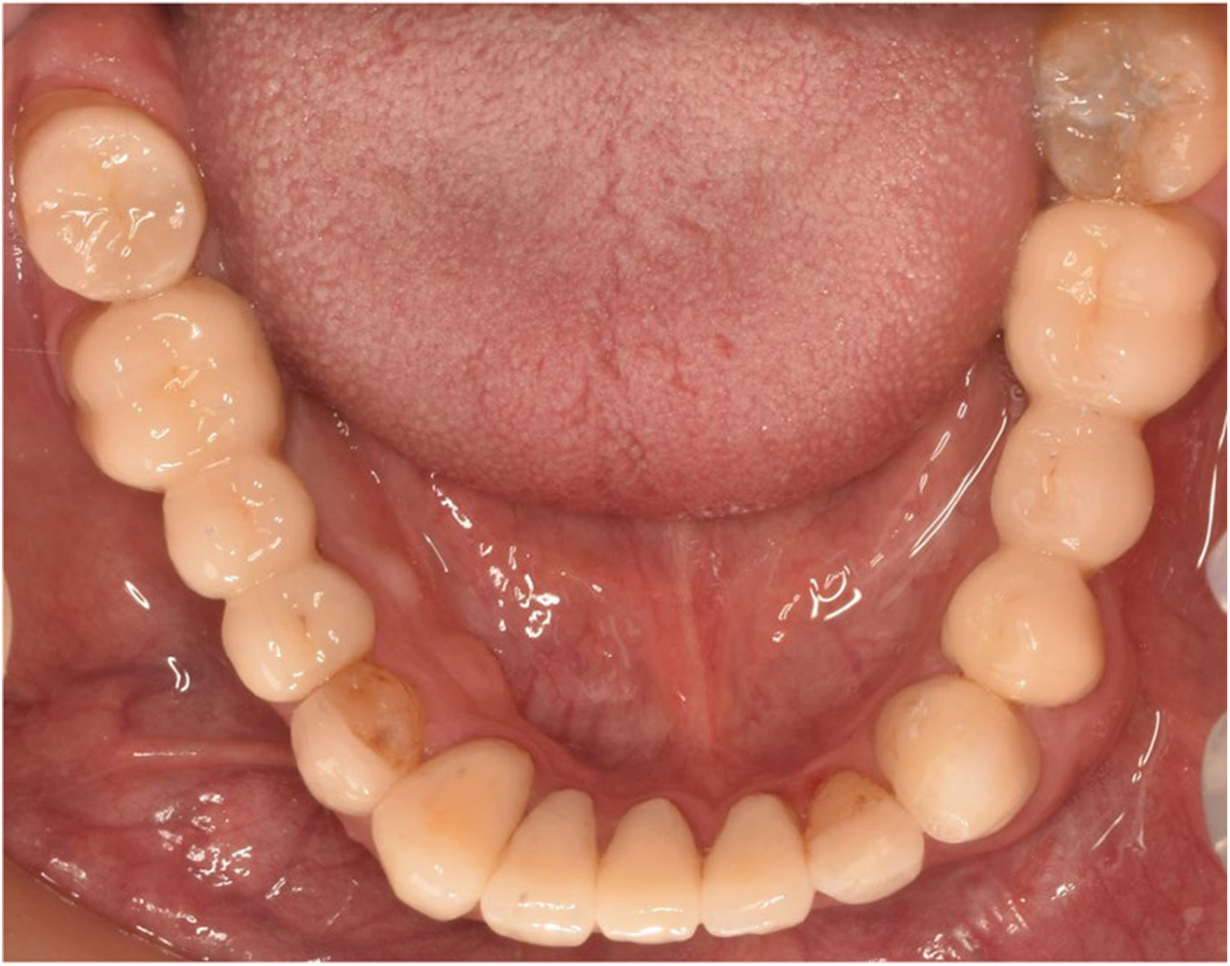
Mandibular occlusal view after cementation 2019.

**FIGURE 8:**
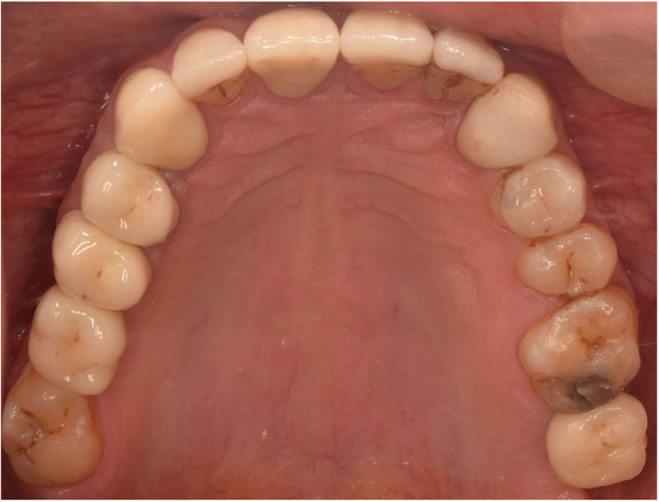
Maxillary occlusal view after cementation 2019.

**FIGURE 9:**
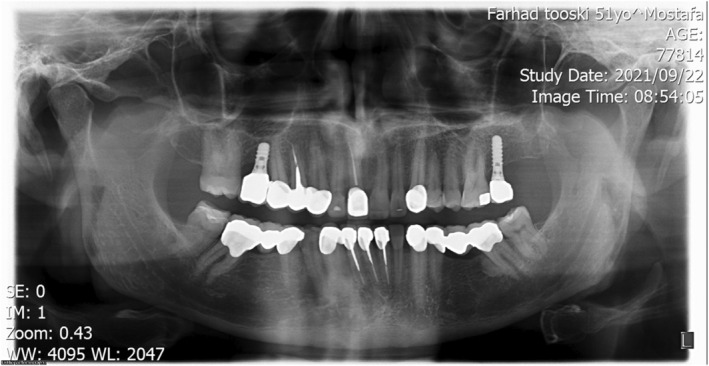
Final panoramic view after delivery 2019.

In the delivery session, laminates were cemented by Choice 2 Veneer Cement (BISCO Dental, USA). Dual‐cure resin cement (Panavia V5, Kuraray Co) was used for occlusal veneers, full coverage restorations cemented by glass ionomer cement (Fuji II, GC Dental Corp), for implant restorations abutments were torqued to 35 N according to manufacturer instruction and temp bond (NE kerr S.R.I. Scafatia, Italia) was used (Figures 6, 7, 8, 9).

**FIGURE 10:**
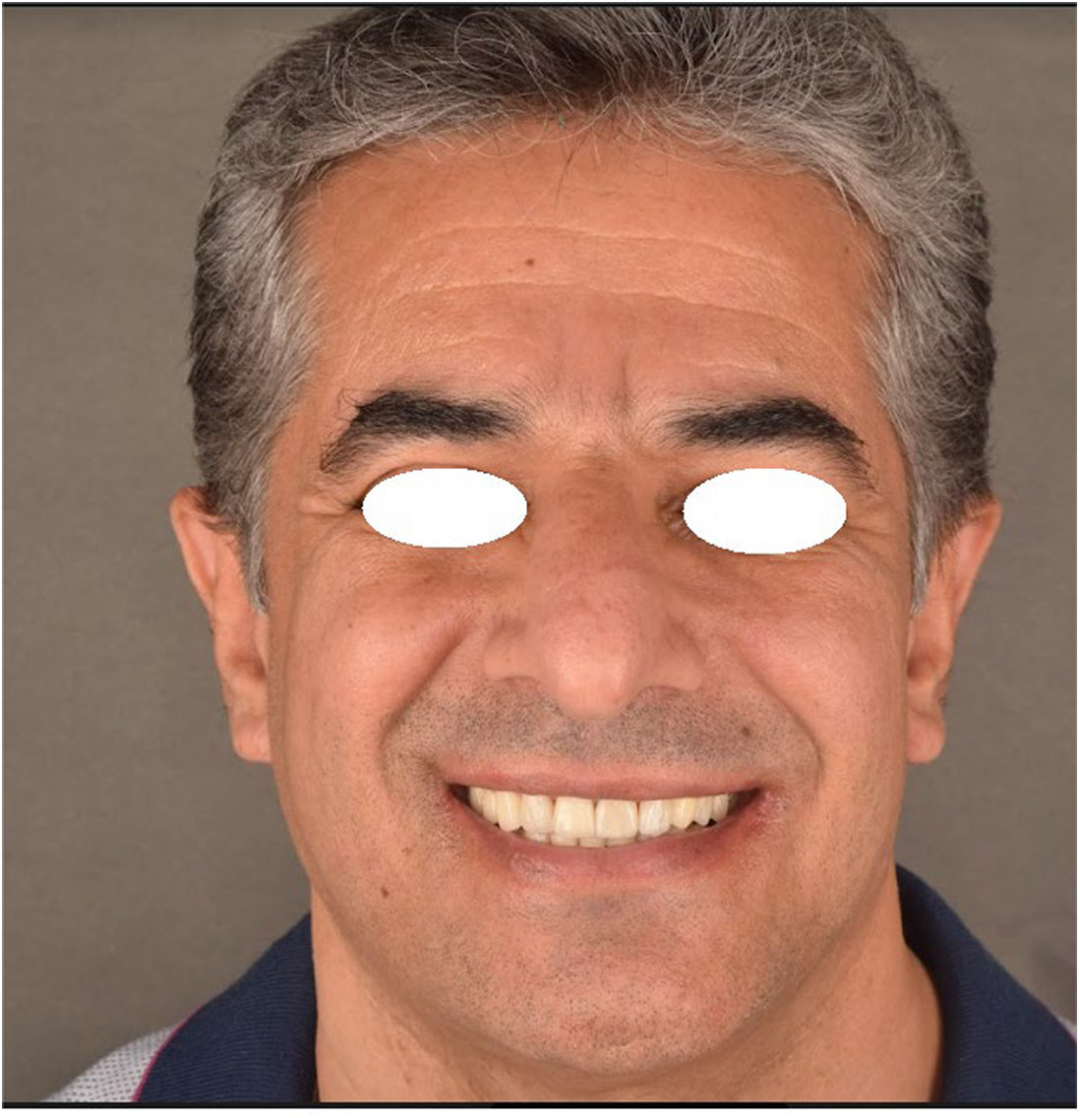
Profile view of a patient after delivery 2019.

**FIGURE 11:**
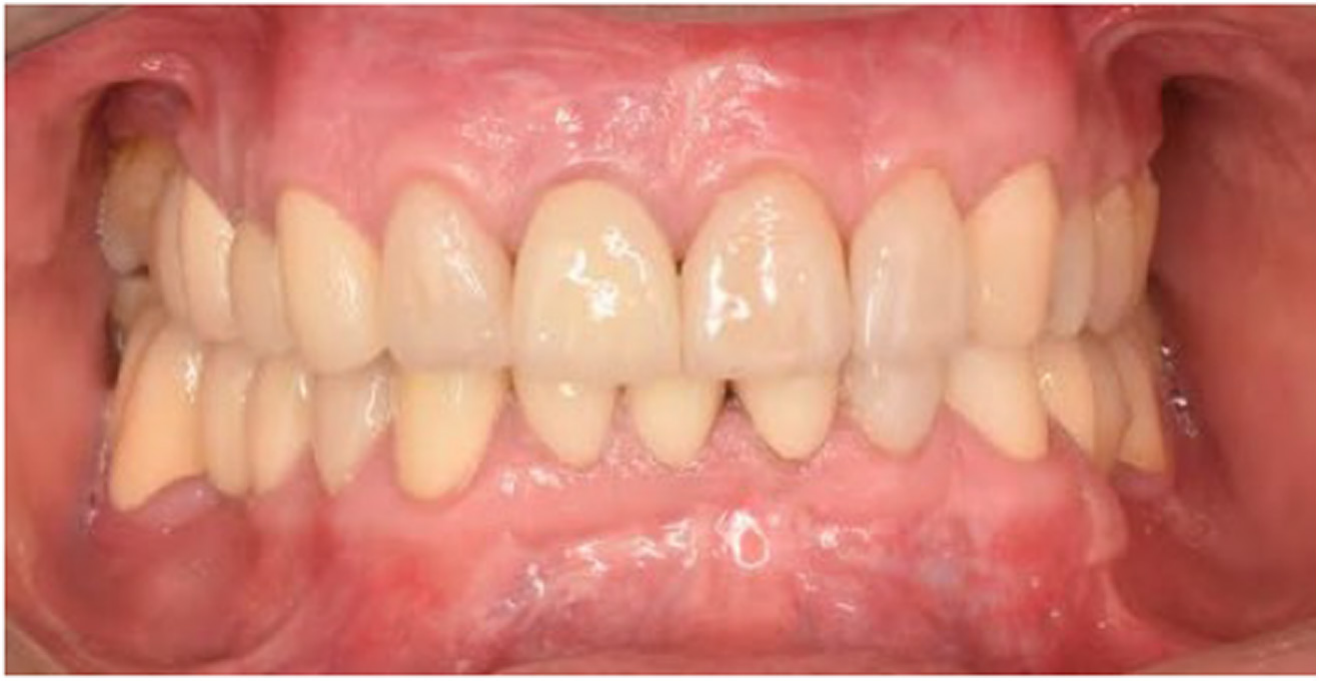
Intraoral frontal view after 3 years follow‐up 2022.

After delivery of restorations, oral hygiene instructions using water jet and super floss was explained for the patient, and follow‐up sessions were set for 1, 6, and 12 months later, and annually afterward. After that an impression (Alginate, Chromogel) was taken for making occlusal dual splint. Delivery of occlusal splint was done in next session.

## 3 DISCUSSION

The case that was presented focused on the treatment procedure of a patient suffered from progressive wear and parafunctional habits. Attempts were made to treat this patient with a conservative approach by minor periodontal surgery which reduced patient's cost and also by considering treatment in his current vertical dimension the risk of future problems following by increasing vertical dimension was decreased. Furthermore, minimally invasive approach by using restorations which involved only one surface (veneers and occlusal onlays) in combination with full crowns using new high strength ceramic materials allowed minimum possible removal of tooth structure.

The challenges in this specific case were anterior deep bite, severe worn teeth, the need of esthetic, and functional rehabilitation in the existing vertical dimension due to patient's intolerance of increasing VD and also the resultant interference of elongated maxillary incisors following with phonetics and lower smile line. It is also challenging to achieve good alignment and occlusal harmony within abutments that have different natures (tooth abutments and implant abutments) simultaneously in full‐mouth rehabilitation with dental implants. Moreover, material selection is a critical factor that needs special attention in worn dentitions since minimal preparation must be performed on the teeth, as well as materials that have sufficient strength in a low thickness must be chosen.

Another challenge was the need to rehabilitate some areas by implant prostheses due to the reported potential failure risk of implant prostheses because of occlusal overload^20^, ^21^, ^22^; however, few researchers have assessed the impact of bruxism on dental implant outcome, and the results obtained are contradictory.^22^, ^23^, ^24^ At present, there are some expert opinions and cautionary approaches that should be considered in order to minimize the risk of implant failure in cases of bruxism.^22^ In this case, zirconia crown was selected for rehabilitating due to its superior esthetics and wear resistance.^25^ Based on studies, an appropriate occlusal scheme (Canine‐guided occlusal concept to reduce occlusal forces during jaw movement) was selected and stable contacts and an equal distribution of forces were established.^22^, ^26^

It is obvious that the loss and wear of the posterior teeth will cause a deeper overbite.^27^ There are several options for gaining space necessary for restorations in these patients who have attrition combined with a deep bite, including restorative dentistry, orthodontics, and oral surgery.

Dawson recommended the following methods for correcting deep bites: (1) Reshaping of anterior teeth as needed in mild cases (2) Orthodontics (3) Restorative procedures (4) Surgery.^5^ There are some cases in which increasing VDO is considered to provide enough space for restorations and to reduce anterior overbite. An alteration in VDO may cause adaptable reactions in the temporomandibular joint (TMJ), periodontium, and occlusal morphology.^28^ By contrast, previous studies have reported that increasing VDO during restorative procedures could be harmful to patients, disrupting their dental physiology and adaptability.^29^, ^30^ Hyperactivity of the masticatory muscles, elevation in occlusal forces, bruxism, and temporomandibular disorders (TMDs) are reported as consequences of increasing the VDO from the literature reviews.^26^, ^27^, ^31^ The VDO should not be increased in cases such as full occlusal rehabilitation where restorative space can be created by crown lengthening or reshaping the teeth.^31^ It should be mentioned that consult with orthodontist and surgical team considering the advantages and disadvantages of orthosurgical treatment and also the time and cost‐effectiveness of the treatment was done and, in the end, it was concluded that optimal treatment result could be obtained just by prosthetic approach. In the following, information was given to him and he preferred and insisted to do the treatment just by prosthetic approach. In the present case, after apace analyzing, gaining space by crown lengthening and restorations was done.

The most important goal of treatment is to form stable occlusal contact in centric relation. The concept of minimally invasive dentistry in appropriate cases preserves dentitions and supporting structures; it has also positive effects on patients' attitude who are impressed by conservative approaches.^28^ Consequently, in this case, stable occlusal contacts were provided for some intact teeth (#2, #13, #14, and #17) since they were in appropriate contour and position. It was decided to prepare lithium disilicate laminates as thin as possible (0.3–0.5 mm) for teeth (#7, #9, #10, and #23). Occlusal veneers, which are considered minimally invasive procedures, were considered for teeth # 12 and # 28. Ceramic occlusal veneers are also known for their superior abrasion and wear resistance, biocompatibility, color stability, and low amount of preparation needed, which is confined to 1 mm.^24^ Planar straight bevel occlusal veneer preparation was considered for occlusal veneers in light of its desirable fracture resistance, decrease in the amount of enamel reduction, favorable fracture load, and lowest maximum principal stress.^29^, ^30^ It should be mentioned that in the final result of treatment discrepancy between upper and lower dental midline is seen which phenomenon by considering the opinion that matching of the maxillary dental midline with the facial midline is more important for the better esthetic outcomes compared with the coincidence of mandibular dental midline with the facial midline and also according to a study prevalence of the maxillary and mandibular midline coincidence in only one fourth of the population and the upper and lower dental midline discrepancy occurs naturally deviated towards one side of the face, and in the case of deep bite (less show of mandibular incisors and midline) and refusal of orthodontic treatment by regarding minimally invasive approach, is inevitable.^32^, ^33^

Finally, the patient's chief complaints were effectively resolved, and he was satisfied with the functional and aesthetic outcomes of his treatment. After 3 years, no evidence of bone loss or loss of VDO and no signs of TMD were noted. The restorations had no signs of chipping or porcelain fracture or wear, and all the surfaces were smooth (Figures 10,, 11). The reason for intact restorations could be informing patient about his parafunctional habit and using occlusal splint to control his bruxism and also using treatment approach in which we did not increase the vertical dimension and also not changing the muscle length.

## 4 CONCLUSION

Full‐mouth rehabilitation requires the proper interdisciplinary concepts to achieve acceptable functional and aesthetic results. It is important to note that the present case report emphasized the steps and phases of the treatment process as well as the use of existing VDO for meeting the biologic, restorative, and esthetic requirements.

## AUTHOR CONTRIBUTIONS

**Negin Yaghoobi:** Methodology; writing – review and editing. **Azam Mostafavi:** Conceptualization; supervision; writing – original draft. **Solmaz barati:** Investigation; writing – original draft; writing – review and editing.

## CONFLICT OF INTEREST STATEMENT

The authors have no conflict of interest in this study and certify that they have no affiliations with or involvement in any organization or entity with any financial interest, or non‐financial interest in the subject matter or materials discussed in the manuscript.

## CONSENT

Written informed consent was obtained from the patient to publish this report in accordance with the journal's patient consent policy.

## Data Availability

The data used to support the findings of this study are available from the corresponding author upon request.
